# Deep Learning Frameworks for Rapid Gram Stain Image Data Interpretation: Protocol for a Retrospective Data Analysis

**DOI:** 10.2196/16843

**Published:** 2020-07-13

**Authors:** Hee Kim, Thomas Ganslandt, Thomas Miethke, Michael Neumaier, Maximilian Kittel

**Affiliations:** 1 Heinrich-Lanz-Center for Digital Health Medical Faculty Mannheim Heidelberg University Mannheim Germany; 2 Institute of Medical Microbiology and Hygiene Medical Faculty Mannheim Heidelberg University Mannheim Germany; 3 Institute for Clinical Chemistry Medical Faculty Mannheim Heidelberg University Mannheim Germany

**Keywords:** high performance computing, rapid Gram stain classification, image data analysis, deep learning, convolutional neural network

## Abstract

**Background:**

In recent years, remarkable progress has been made in deep learning technology and successful use cases have been introduced in the medical domain. However, not many studies have considered high-performance computing to fully appreciate the capability of deep learning technology.

**Objective:**

This paper aims to design a solution to accelerate an automated Gram stain image interpretation by means of a deep learning framework without additional hardware resources.

**Methods:**

We will apply and evaluate 3 methodologies, namely fine-tuning, an integer arithmetic–only framework, and hyperparameter tuning.

**Results:**

The choice of pretrained models and the ideal setting for layer tuning and hyperparameter tuning will be determined. These results will provide an empirical yet reproducible guideline for those who consider a rapid deep learning solution for Gram stain image interpretation. The results are planned to be announced in the first quarter of 2021.

**Conclusions:**

Making a balanced decision between modeling performance and computational performance is the key for a successful deep learning solution. Otherwise, highly accurate but slow deep learning solutions can add value to routine care.

**International Registered Report Identifier (IRRID):**

DERR1-10.2196/16843

## Introduction

In recent years, remarkable progress has been made in deep learning due to the emergence of big data processing technology. Deep learning is a family of machine learning that consists of multiple neurons in multiple layers. A neuron is a mathematical function with weights and biases, known as parameters. It receives real numbers from the neurons in the previous layer, generates another real number, and transmits it to the neurons in the next layer. The parameters for each of these neurons are optimally determined by a backpropagation algorithm, such as stochastic gradient descent, that looks for the minimum of a function. This contributes to the success of deep learning of image data compared with conventional techniques because it is able to learn the intrinsic data features without handcrafted feature engineering.

Gram stain is a laboratory procedure for the rapid classiﬁcation and identiﬁcation of microbial pathogens. Unlike other microbiology processes that can be fully automated [1], the interpretation of Gram stain images still relies on human users, such as a physician or trained medical technical assistant. Although Gram stain seems like a medical image analysis problem, to the best of our knowledge, only 1 research paper [2] has used the deep learning method to automate the Gram stain analysis. One of the challenges is that microbial pathogens, particularly gram-negative organisms, and the background material, such as bloodstains, look highly similar on a slide. Furthermore, in cases of a low density of bacteria in a clinical sample, the manual search by microscopy is tedious and will only examine a fraction of the microscope slide, and thus may be error-prone.

Smith et al [2] achieved 94.9% classification accuracy out of 468 Gram-stained slides from positive blood cultures. The respective sensitivities and specificities were 98.4% and 75.0% for gram-positive cocci in chains and pairs, 93.2% and 97.2% for gram-positive cocci in clusters, and 96.3% and 98.1% for Gram-negative rods. The authors reused the pretrained model called Inception-v3 [3] and retrained the last layer with their image data (100,213 image crops of 146×146 pixels for training and testing) instead of constructing an end-to-end model from the scratch. This approach is called transfer learning [4] because it reuses precomputed model parameters. The major advantage of transfer learning is that it is able to reduce computational costs for the model training.

Despite the high accuracy achieved by Smith et al, there are still many open questions to be addressed. With regard to modeling, transfer learning could be improved with fine-tuning [5] instead of modifying only the last layer. Fine-tuning is a type of transfer learning that allows one to adjust the ratio of retraining layers. This is highly relevant to Gram stain classification because Inception-v3 was constructed with ImageNet [6], which contains 1.2 million nature images and 1000 image classes, such as dogs and cats, that are unrelated to Gram stain images. Therefore, increasing the number of unfrozen (retraining) layers (ie, decreasing the number of freezing layers) with Gram stain images is anticipated to yield better results. With respect to computational performance, it takes about 9 minutes to classify a whole-slide image of 28,032×28,032 pixels with a computer consisting of Intel Core i7 (Intel Corp) with 32 GB of RAM and a Nvidia GTX 1070 graphics processing unit (GPU). The turnaround time for multiple samples encountered in the medical laboratory would not provide timely decision-making, although this solution can run the job 24/7.

This study aims to design a rapid deep learning solution for Gram stain interpretation without acquiring hardware resources, and it provides the optimal proportion for the fine-tuning. The hypothesis and the study design to evaluate the hypothesis will be explained in the following section.

## Methods

This section addresses the hypothesis, study design, data collection and description, study population, statistical considerations for nonbiased model construction and evaluation, and tools in detail.

### Study Design

**Figure 1:**
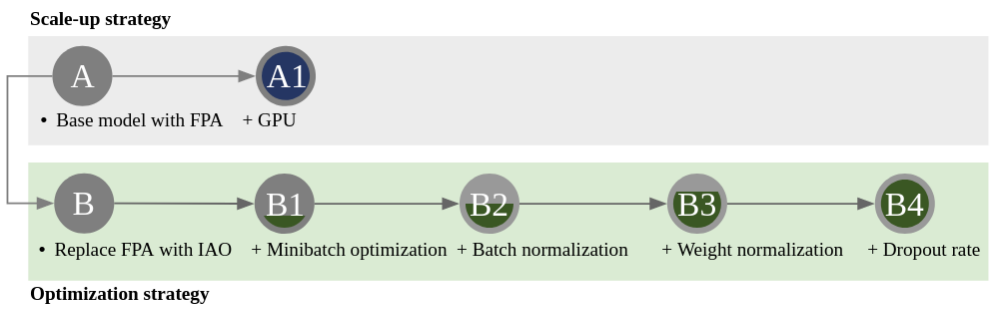
Two strategies will be compared. The lineage of model A is the implementation of the scale-up strategy (highlighted in blue). On the other hand, the lineage of model B is the implementation of the optimization strategy (depicted in green). Model A is the base model with FPA framework, while model B replaces the floating-point arithmetic with IAO. Each model is built on top of a predecessor model. For instance, model A1 is empowered with a single GPU and model B1 is empowered with the optimal minibatch size. FPA: floating-point arithmetic. GPU: graphics processing unit. IAO: integer arithmetic only.

This study does not investigate a clinical hypothesis but performs an empirical evaluation of a deep learning framework for Gram stain image interpretation. The hypothesis to be tested is that the optimization of a deep learning framework will perform better than a scale-up strategy with a single GPU. In order to test this hypothesis, two strategies will be examined, as shown in Figure 1. The scale-up strategy stacks up more computer capabilities (model A, highlighted in blue). On the other hand, the optimization strategy tunes the granular configuration of a deep learning framework (model B, depicted in green).

In order to avoid model bias, 4 pretrained models (Mobilenet [7] with versions 1 and 2, and Inception [3] with versions 3 and 4) will be evaluated. Mobilenet and Inception were originally trained with ImageNet [6], which contains 1.2 million nature images and 1000 classes. Therefore, it is necessary to retrain them on the new data set, as described in the “Data Collection” section, because Gram stain interpretation is barely linked to the original task. The method that retrains a pretrained model without changing network architecture is called fine-tuning. Fine-tuning is a specific transfer learning technique that modifies more layers than the last layer, also known as the fully connected layer, and their corresponding weights and biases. The optimal proportion of the fine-tuning will be empirically determined in this study. Concretely, 10 implementations will be evaluated, with the proportions of frozen layer to unfrozen layer (retraining layer) at 9:1, 8:2, ongoing up to 0:10 (from the shallow strategy to the deep strategy). The computational performance will be measured by time to achieve the target accuracy metric [8] of 95%.

Once the base model is implemented with Tensorflow [9] (Google Corp) as described above, model A will be accelerated with 2 approaches. Model A1 is the scale-up implementation built on top of model A with a single GPU. In contrast, model B is a mutated model of model A because it replaces floating-point arithmetic with an integer arithmetic–only deep learning framework called Tensorflow Lite [10]. Model B1 aims to detect the optimal batch size for Gram stain classification. Model B2 and B3 activate batch normalization and weight normalization. Normalization penalizes large input numbers and weights. Finally, model B4 prunes the model network by adjusting the dropout rate, which speeds up the execution time. These hyperparameters—batch size, batch normalization, weight normalization, and dropout rate—are chosen based on the findings from previous studies [11-14]. The specification of the hyperparameter space is defined in Table 1. The boundary is chosen to be wide to account for many possible combinations of the hyperparameters.

**Table 1:** Specification of the hyperparameter space for the model B family. Minibatch size and dropout rate are quantified to avoid an exhaustive search.

Model	Hyperparameter	Original value range	Quantified values
B1	Minibatch size	{1-infinity}	{32, 64, 128, 256, 512}
B2	Batch normalization	{on, off}	{on, off}
B3	Weight normalization	{on, off}	{on, off}
B4	Dropout rate	{0-1}	{0, 0.1, 0.2, 0.3, 0.4, 0.5}

The objective of this study is to understand the relation between computation time and each hyperparameter, not to create a hyperparameter optimization [15] that searches for a global minimum or the local minima of hyperparameters.

### Data Collection

**Figure 2:**
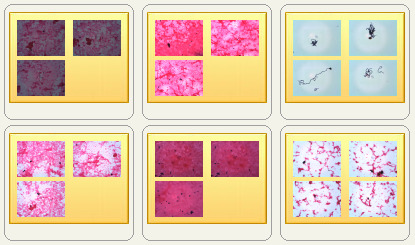
A sample image of Gram stain data. The image label does not have a link to personal information.

This study will use 8728 Gram stain images from between 2015 and 2018 for modeling and images generated in 2019 for testing. Data are archived in a workstation at the Institute for Clinical Chemistry at the Medical Faculty Mannheim of Heidelberg University, Germany. Sample images and labels are shown in Figure 2. The sizes of the cropped images vary from 800×600 pixels to 1920×1080 pixels. Note that the image is not a whole-slide image, but a crop of the interests—the microorganisms and the slide background.

The label data corresponding to the image are stored in a central database for reporting purposes and extracted for this study. Each image is associated with 2 labels: (1) Gram stain class (ie, either gram-positive or gram-negative) and (2) a class for the genus. The genus label includes 5 of the most frequently encountered germs: (1) *Staphylococcus*, (2) *Escherichia*, (3) *Streptococcus*, (4) *Enterococcus*, and (5) others, for the rest of the germs that are rarely presented. This setting prevents a potential risk of identifying a patient with an extremely rare microorganism. Of the 8728 images, 446 images (5.11%) are associated with multiple classes. These images with multiple class labels are excluded because object recognition, that is, bacterial differentiation, is beyond the scope of this study.

### Study Population

The population for this study is a group of sepsis patients, whose blood samples contain at least one harmful bacterium, such as *Staphylococcus*, *Escherichia*, or *Streptococcus*. This study does not recruit control and treatment groups. However, a set of gram-positive images would be regarded as one group, while gram-negative images would be regarded as a comparison group for this study. This is a retrospective data analysis reusing an archived image data set, as described in the “Data Collection” section.

### Statistical Analysis

This section will address and describe the 3 underlying statistical considerations towards a solid study design: (1) the class balancing strategy for the input data set, (2) the proper split ratio for training and evaluation, and (3) the metric for the model evaluation.

#### Class Balancing

Imbalanced input data sets are a common limiting factor that degrades model quality. Chawla et al [16] systematically proved that data augmentation can improve the imbalanced class problem and demonstrated the benefits. In the classification of bone lesions from x-ray images, Gupta et al [17] mitigated the small number of positive samples by using data augmentation. In the given image data available for this study, gram-positive results are twice as frequent as gram-negative results. In order to balance the class proportion, this study will apply the data augmentation technique, which enriches the data set by cropping, rotating, zooming, and ﬂipping the given images.

#### Split Ratio

The data set will be split into a training set, a hold-out development set, and a test set. The hold-out development set is different from the test set, as the development set will only be used for tuning the model parameters in order to not bias classification. The training set for deep learning algorithms is increased to 99% of the entire data set when there are more than a million data points. However, this study will follow best practice in machine learning, in which the splitting ratio is 60%, 20%, and 20% [18], because the available data points for this study are 8728 images.

Cross-validation is not used in this study for model validation. Cross-validation estimates the performance of the model statistically, but it is not the chosen method for evaluating a deep learning model. For instance, a 10-fold cross-validation creates a model with 9 folds and tests the model with the hold-out data (1 fold) 10 times. When we evaluate the model with 100 whole-slide (28,032×28,032 pixels) images, each round will take at least 900 minutes with a workstation powered by Intel Core i7 with 32 GB of RAM and a Nvidia GTX 1070 GPU, which is the same hardware setting and the same image size used in the study by Smith et al [2]. For the 10-fold cross-validation, it would take more than 6 days to evaluate 1 model, which is beyond the capacity of the workstation.

#### Metric for Evaluation

Despite considerable efforts that have been devoted to deep learning research, not many studies consider the computational efficiency, but focus solely on model evaluation. In order to provide more insightful information, this study will evaluate models with the classical metrics, such as accuracy, confusion matrix, and area under curve, as well as the training and testing times of models, to achieve the target accuracy proposed by the Stanford Data Analytics for What’s Next project team [8].

### Apparatus

This study will use Tensorflow [9] (floating-point arithmetic framework) and Tensorflow Lite [10] for deep learning solutions. Both of these frameworks are open source tools developed by the Google Brain team, and they are able to accelerate deep learning calculation by using multicore central processing units (CPUs) and GPUs. With regard to a model-debugging tool, TensorBoard will be used to graphically track all execution history.

All solutions will be developed and deployed in the data center at the Heinrich-Lanz-Center for Digital Health. The hardware configuration for this study is one Intel Xeon Silver 4110 CPU (Intel Corp), one Tesla V100 PCIe 32 GB GPU (Nvidia Corp), and 189 GB memory. The server is virtualized by Docker technology (Docker Inc) [19] for reproducible research.

## Results

This study will provide an empirical guideline on how to accelerate a high-performance deep learning model without losing predictive power. Concretely, 2 results will be highlighted: (1) the performance improvement of an integer arithmetic–only deep learning framework for Gram stain image classification and (2) the optimal setting of fine-tuning and hyperparameters for 4 pretrained models (Mobilenet version 1 and 2 and Inception version 3 and 4). All models and the code for training and evaluation will be freely accessible in a public repository for reproducible research.

As of October 2019, this study has been approved by the institutional review board of Medical Faculty Mannheim of Heidelberg University, and the image data for the retrospective data analysis are available. The results are planned to be announced in the first quarter of 2021.

## Discussion

### Limitations

Distributed computing across multiple machines will not be covered in this study. Although it is the usual method to process big data, it is not always the most efficient choice to process the data. According to Boden et al [20], distributed systems are surprisingly inefﬁcient in training machine learning models compared with a single workstation. The size of the input data for this study is 25 GB and it fits into the capacity of a standalone workstation. In future work, we would like to study Apache Spark [20], which enables distributed machine learning model training when the data do not fit into the memory of a single computer.

This study does not aim to propose novel neural network architecture, which requires many days of GPU processing time with state-of-the-art computational infrastructure that is not available within the scope of this project. Also, designing an outperforming architecture for image classification is a saturated topic, as many researchers have devoted their endeavors to this problem in the last decade. Nevertheless, for those who are interested in this topic, Elsken et al published a state-of-the-art review paper [21] that provides an overview of existing works and categorizes them into 3 dimensions.

### Risk of Project Failure

An insufficient amount of image data could lead to an underpowered deep learning solution. The proper input data size is still an open question in the computer vision community. The answer is, “it depends.” It depends on the number of classes, image size, image quality, and complexity of the problem. For instance, classifying a black image versus a white image demands fewer input data compared with classifying a gram-positive image versus a gram-negative image.

In medical data analysis, power analysis is widely applied for determining the minimum sample size required. Unfortunately, power analysis is not applicable to unstructured data such as images. A rule of thumb for a good input size is 1000 images per class [21], which was the basis of an object recognition competition that was part of the Pascal Visual Object Classes challenge [22]. This study is anticipated to have low failure, since about 5800 gram-positive labeled images and 2700 gram-negative images are available for modeling.

### Data Protection Considerations

Although this study does not use any personal information for data analysis, the name of the input data consists of a unique identifier for the experiment. This experiment identifier harbors a remote risk of linking back to personal information in the database. In the interest of data protection, this identifier is anonymized and securely stored at the Heinrich-Lanz-Center for Digital Health , which is protected by the hospital network ﬁrewalls. Unlike data pseudonymization, which transforms the identifier, data anonymization is an irreversible technique that removes the identiﬁer permanently. The anonymized data will be archived for reproducibility.

The study will comply with the latest version of the Declaration of Helsinki [23] and Professional Code for Physicians in Germany. Patient names and other personal data are subject to the legal requirements concerning conﬁdential medical communication. They comply with European Directive 2016/679 of the European Parliament and of the Council of 27 April 2016 on the protection of individuals with regard to the processing of personal data and on the free movement of such data, the EU General Data Protection Regulation and the German Federal Data Protection Act, and the State Data Protection Act Baden-Württemberg.

## Abbreviations

CPU: central processing unit
GPU: graphics processing unit
